# Regulation of SIRT1 in Ovarian Function: PCOS Treatment

**DOI:** 10.3390/cimb45030133

**Published:** 2023-03-02

**Authors:** Xinrong Li, Yuxu He, Shuang Wu, Peiwen Zhang, Mailin Gan, Lei Chen, Ye Zhao, Lili Niu, Shunhua Zhang, Yanzhi Jiang, Zongyi Guo, Jinyong Wang, Linyuan Shen, Li Zhu

**Affiliations:** 1 Department of Animal Science, College of Animal Science and Technology, Sichuan Agricultural University, Chengdu 611130, China; 2 Farm Animal Genetic Resource Exploration and Innovation Key Laboratory of Sichuan Province, Sichuan Agricultural University, Chengdu 611130, China; 3 College of Life Science, Sichuan Agricultural University, Chengdu 611130, China; 4 Chongqing Academy of Animal Science, Rongchang, Chongqing 402460, China

**Keywords:** SIRT1, ovary, follicle development, granulosa cells, research progress

## Abstract

The sirtuin family, a group of NAD+-dependent class 3 histone deacetylases (HDACs), was extensively studied initially as a group of longevity genes that are activated in caloric restriction and act in concert with nicotinamide adenine dinucleotides to extend the lifespan. Subsequent studies have found that sirtuins are involved in various physiological processes, including cell proliferation, apoptosis, cell cycle progression, and insulin signaling, and they have been extensively studied as cancer genes. In recent years, it has been found that caloric restriction increases ovarian reserves, suggesting that sirtuins may play a regulatory role in reproductive capacity, and interest in the sirtuin family has continued to increase. The purpose of this paper is to summarize the existing studies and analyze the role and mechanism of SIRT1, a member of the sirtuin family, in regulating ovarian function. Research and review on the positive regulation of SIRT1 in ovarian function and its therapeutic effect on PCOS syndrome.

## 1. Introduction

The sirtuin family is principally involved in the regulation of post-transcriptional modifications and belongs to a group of histone deacetylases [1]. In bacteria, yeast, and mammals, sirtuins have similar catalytic core regions and are highly conserved across species. Sirtuins were first recognized as longevity genes [1], and it was reported that the overexpression of Sir2 could extend the lifespan of yeast by up to 70% [2]. Subsequent studies also revealed that sirtuins can bind to the p53 protein, deacetylate it, and disable the transcription factor activation of its C-terminal lysine residue, thereby reducing the transcriptional activity of p53, and thus unveiling the sirtuin family’s mechanism of action [3]. In addition, the sirtuin family is involved in aging, caloric restriction, and age-related diseases, and responds to DNA damage, oxidative stress, and energy metabolism by activating or inhibiting AMPK, mTOR, and other signaling pathways via the deacetylation-based regulation of various genes [4,5]; these include the tumor suppressor gene p53, nuclear factor-KB (NF-κB), peroxisome proliferator activated receptor y coactivator 1-α (PGC-1α), FOXO, liver x receptor (LXR), PARP, Ku70, and hypoxia-inducible factors. Thus, the sirtuin family participates in various physiological processes, including cell proliferation, apoptosis, cell cycle progression, and insulin signaling [2,6].

Sirt1, the human homologous sirtuin family gene of *Saccharomyces cerevisiae* Sir2, is the most studied and well-characterized sirtuin and was initially studied extensively as a longevity gene. Previous studies have shown that SIRT1 can act to delay the cellular lifespan by deacetylating the p53 protein and reducing its transcriptional activity. It has also been found that Sirt1 maintains essential biological functions through the role of Ku proteins bound to DNA ends, which are involved in the repair of broken DNA ends during DNA damage repair [7]. Subsequently, Sirt1 has been widely studied as a cancer gene because of its unique role in transcriptional silencing, cell cycle progression, and chromosome stability. In addition, Sirt1, as a NAD+-dependent deacetylase, plays a regulatory role through the deacetylation of downstream proteins and shows high expression in mammalian ovarian follicles, which is dynamically associated with different processes of follicle development; thus the regulatory role of Sirt1 in ovarian tissues has attracted much attention [8].

Sirtuins directly link cellular metabolic signals to the post-translational modification of proteins, and the mechanism of action is mediated by the transfer of acetyl groups to the ADP-ribose fraction to form O-acetyl-ADP-ribose (O-acetyl-ADP-ribose, OAADPr), and the release of free nicotinamide (nicotinamide, NAM). Depletion of NAD+ by acetylation combines the deacetylation of lysine with the hydrolysis of NAD+ and is accompanied by the production of deacetylated proteins [9]. Since this reaction requires NAD+ and the NAD+/NADH ratio is determined by the trophic state of the cell, the catalytic activity of sirtuins is regulated by the level of nicotinamide transphosphate ribokinase (NAMPT) in the cell and by changes in the NAD+ concentration or NAD+/NADH ratio. Sirtuins not only deacetylate histones, but also regulate a wide range of transcriptional events, thereby controlling their activity [10].

Although most members of the sirtuin family exert regulatory effects through deacetylation, there are significant differences in their subcellular localization and the regulatory functions involved. In mammals, the sirtuin family consists of seven proteins (SIRT1-SIRT7) that differ in tissue specificity, subcellular localization, enzymatic activity, and targeting. Mammalian sirtuin proteins display a discrete pattern of subcellular localization. SIRT1 is primarily localized to the nucleus, but its nuclear export signal allows shuttling into the cytoplasm under certain circumstances; SIRT2 is primarily present in the cytoplasm, but may migrate into the nucleus when cells are in the G2 to M phase transition of the cell cycle; SIRT3, SIRT4, and SIRT5 have mitochondrial targeting sequences and their localization to this organelle has been experimentally confirmed; SIRT6 and SIRT7 are mainly present in the nucleolus, but further studies are needed to analyze their localization [11].

**Figure 1:**
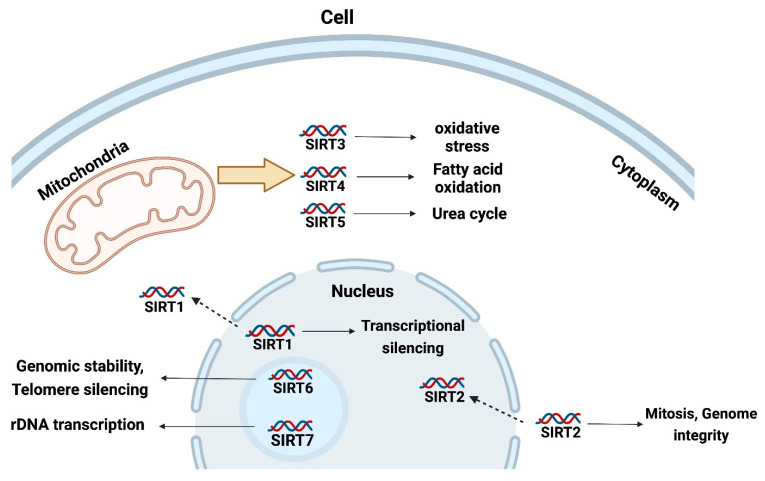
Main functions and subcellular localization of sirtuin family members.

In addition, different members of the sirtuin family have been shown to regulate different biological activities. According to the literature, SIRT1 mainly regulates transcriptional silencing, mitochondrial regulation, insulin signaling, tumorigenesis, apoptosis, cell proliferation and survival, tissue regeneration, differentiation, and the stress response; SIRT2 is involved in mitosis, neuromyelin formation and regeneration, brain aging, and adipocyte differentiation; SIRT3 regulates fatty acid oxidation, the TCA cycle, oxidative phosphorylation, and oxidative stress; SIRT4 regulates the TCA cycle and fatty acid oxidation; SIRT5 is involved in the urea cycle; SIRT6 is involved in genome stability and telomere silencing; and SIRT7 regulates rDNA transcription. To date, the regulatory activities of SIRT1 and SIRT2 have been more thoroughly explored, and the mechanisms of action and the biological activities of the other sirtuin family members require further study [11,12] (as shown in Figure 1).

## 2. Regulation of Granulosa Cell Proliferation, Apoptosis, and Steroid Synthesis by SIRT1

### 2.1. Granulosa Cell Proliferation, Apoptosis, and Luteinization

Ovarian granulosa cells surround the primordial follicle and participate in follicular development, maturation, and the ovulation process, and can be divided into two types according to the location of the follicle that they are in and the biological function that they perform. One type is the oocyte granulosa cell, which is tightly wrapped around the oocyte, while the other is the wall granulosa cell, which is immediately adjacent to the follicle wall. Mural granulosa cells mainly support the development of the oocyte and participate in the regulation of the follicular maturation process, whereas the main function of mural granulosa cells is to act as hormone receptors, including follicle-stimulating hormone (FSH) receptors and luteinizing hormone (LH) receptors. After ovulation, the mural granulosa cells are gradually transformed into luteal cells and participate in a subsequent series of hormonal regulation processes. Granulosa cells are essential during follicular development and their apoptosis is directly related to follicular atresia, while, in the dominant follicle, they maintain the entire process from follicular development to ovulation by providing an energy supply, endosteroid synthesis, and reactive oxygen species scavenging.

Therefore, the regulation of granulosa cells’ proliferation and apoptosis plays a direct role in determining the fate of the follicle. The finding of high expression of SIRT1 in ovarian granulosa cells in the study of Benayoun et al., and its ability to scavenge reactive oxygen species, indirectly confirms this idea [13]. The regulatory role of SIRT1 in the granulosa cells themselves has also been reported in many studies. Subsequently, Zhao et al. found that the expression of SIRT1 during follicular atresia in porcine ovaries showed dynamic changes along with granulosa cell apoptosis, suggesting that SIRT1 could be involved in the regulation of follicular atresia-related processes mediated by granulosa cell apoptosis in the ovaries [14]. Nie et al. were able to protect against premature ovarian failure induced by cyclophosphamide in a mouse ovarian model by significantly inhibiting the expression of the pro-apoptotic protein Bax and by stimulating the expression of the anti-apoptotic protein Bcl-xL through the activation of SIRT1 by Ras [15]. Han et al. found that SIRT1 increased apoptosis resistance in granulosa cells by activating the ERK1/2 signaling pathway and inhibiting the NF-κB signaling pathway, which has anti-inflammatory functions [16]. Sirotkin found similar results in porcine ovarian granulosa cells, where SIRT1 has been shown to regulate transcription factors p53 and NF-κB, which are involved in the regulation of the apoptosis and proliferation of ovarian granulosa cells [17]. Luo et al. identified differentially expressed miRNAs in patients with different levels of POI; only the abbreviation is described. Szymanska et al. also identified a regulatory role for SIRT1 in granulosa-luteinizing cells. The authors first found that the cAMP pathway promotes SIRT1 expression in human granulosa-luteinizing cells [18], while a subsequent study also found that SIRT1 produces repressive epigenetic modifications through the EDN2 promoter [19]. Zhang et al. found that miR-181a mediates oxidative stress by targeting SIRT1 in vitro and in vivo, resulting in FoxO1 acetylation and granulosa cell apoptosis, suggesting that ROS is also a factor in the induction of granulosa cell apoptosis [20].

SIRT1 can directly regulate the expression of Bax and Bcl-xL, which are involved in the regulation of proliferation and apoptosis in granulosa cells, as well as indirectly, by participating in other signaling pathways. In addition, the energy supply during follicular development is mainly provided by granulosa cells, and the imbalance of ROS metabolism under the high intensity of mitochondrial respiration may indirectly lead to granulosa cell apoptosis, which is blocked by SIRT1. It is thus clear that granulosa cells are essential for ovarian function and follicular development processes, and that SIRT1 participates in their proliferation, apoptosis, luteinization, and other biological processes.

### 2.2. Steroid Hormone Synthesis

**Figure 2:**
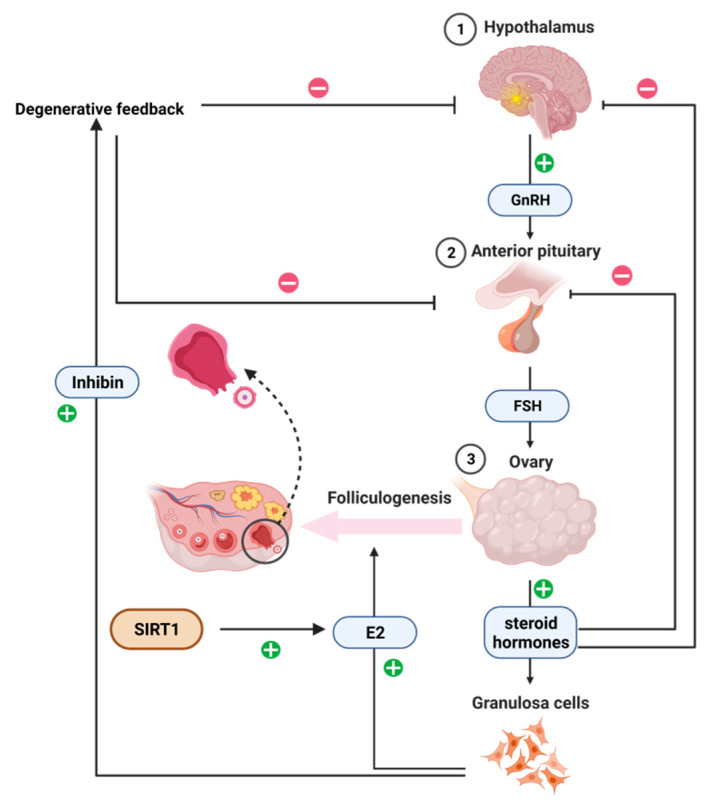
Regulation of steroid hormone synthesis by the hypothalamic–pituitary–ovarian axis.

In the mammalian reproductive system, SIRT1 is thought to contribute to steroid hormone signaling and the physiological control of reproduction. The secretion of gonadotropin (FSH and LH) is stimulated by hypothalamic-derived gonadotropin-releasing hormone (GnRH), which provides the primary mechanism controlling follicle selection and dominance through a feedback loop with the hypothalamic–pituitary–ovarian axis. Furthermore, granulosa cells were observed to be the initial cell population that undergoes apoptosis, i.e., earlier than oocytes and follicular membrane cells in atretic follicles, suggesting that granulosa cells are the initiating cells in follicular atresia. The mechanism is observed in the negative feedback effect of the ovary, where androgens produced by the membrane cells are used by the granulosa cells to synthesize estradiol, while the granulosa cells themselves produce inhibin, which acts in concert with the hypothalamic–pituitary system to reduce the secretion of FSH and thus inhibit further secondary follicle growth. Steroid hormones regulate both the development of the dominant follicle and the process of atresia in the secondary follicle, and studies have shown that SIRT1 regulates the expression of aromatase in estrogen-producing cells through binding to the aromatase promoter and through deacetylation [21]. Thus, SIRT1 is essential for the regulation of steroid synthesis and thus participates in the overall development of ovarian follicles. SIRT1 regulates ovarian steroid hormone secretion through the regulation of granulosa cell function, but additional studies have found that SIRT1 also has other regulatory pathways (as shown in Figure 2).

Brain and muscle ARNT-like protein 1 (BMAL1) is essential for fertility, and its regulation of steroidogenesis has been confirmed by many studies in vivo and in vitro. Zhang et al. found that SIRT1 regulates aromatase expression directly through the positive regulation of BMAL1, while BMAL1, via positive feedback, also elevates the expression of SIRT1, and this feedback mechanism could be how SIRT1 regulates steroid synthesis [21]. Other related transcription factors that regulate SIRT1 are also involved in steroidogenesis. Reverchon et al. found that nicotinamide phosphate glycosyltransferase (NAMPT) increases SIRT1 activity by promoting the biosynthesis of nicotinamide adenine dinucleotide (NAD) and induces steroidogenesis in bovine ovarian granulosa cells [22]. Whereas the abnormal expression of SIRT1 may contribute to endocrine disorders, Dubey found that the ESC/E(Z) complex exhibits intrinsic differences in cells of premenstrual irritable women with abnormal expression of one of its constituent proteins, SIRT1, leading to an abnormal ovarian steroid response [23]. Tae Hoon Kim similarly found that progesterone resistance and abnormally high SIRT1 expression may be involved in the development of pathological symptoms associated with endometriosis [24].

SIRT1 plays an important role in ovarian steroid hormone regulation. Therefore, it is of crucial clinical importance to regulate the homeostasis of ovarian endosteroids through the regulation of SIRT1 expression in the ovaries. For various ovarian endocrine disorders, such as polycystic ovary syndrome (PCOS), metformin, melatonin, and resveratrol have been used as targeted therapies, the underlying mechanisms of which are closely related to the expression of SIRT1, thus achieving significant therapeutic effects.

## 3. Regulation of Oxidative Stress and Mitochondrial Function by Sirt1 in Granulosa Cells

### 3.1. Oxidative Stress

Pyruvate production from the mitochondria of the granulosa cells of the ovarian follicle provides the main energy source during oocyte maturation due to the inadequate capacity of the oocyte to metabolize glucose. High mitochondrial oxidative phosphorylation in granulosa cells leads to the inevitable production of ROS, and high levels of FSH-stimulated mitochondrial oxidative phosphorylation in granulosa cells during follicular development are responsible for excess ROS, leading to oxidative stress [25].

Early in 2008, Benayoun et al. found that FOXL2 is highly expressed in the granulosa cells of fetal and adult ovarian follicles, and its expression is regulated by SIRT1 and significantly upregulated under oxidative stress and through the upregulation of the ROS scavenging enzyme, mitochondrial manganese superoxide dismutase (MnSOD), thus maintaining the ovarian follicular environment [26]. In contrast, SIRT1 was found to play a critical role in the regulation of cellular stress responses and physiological processes, and its knockdown in mice resulted in low fertility and reproductive phenotypes, growth retardation, as well as high perinatal mortality. A subsequent study by the authors revealed the existence of a feedback mechanism between FOXL2 and SIRT1: FOXL2 activates SIRT1 transcription and upregulates its expression through promoter binding, while SIRT1 plays a regulatory role by deacetylating FOXL2, thus maintaining the homeostasis of the ovarian follicle internal environment. A subsequent study by Park et al. also found that H_2_O_2_ induced oxidative stress by inhibiting the SIRT1 upregulation of p53 activity, which led to granulosa cell apoptosis, thus suggesting a mechanistic role of the SIRT1/p53 axis in H_2_O_2_-induced granulosa cell apoptosis [27]. Ding et al. showed that the combined effect of hepatocyte growth factor (HGF) and basic fibroblast growth factor (BFGF), secreted by human adipose stem cells, slowed the natural aging process by activating the SIRT1/FOXO1 signaling pathway, reducing ROS production and oxidative stress [28]. Mihanfar et al. found a significant increase in the expression of AMP-activated kinase (AMPK) and SIRT1, and the related antioxidants catalase (CAT) and superoxide dismutase (SOD), as well as a decrease in the expression of CYP17A1, in a rat model of PCOS treated with fexarone. This suggests that SIRT1 may act via crosstalk with other pathways to play a potential role in the synthesis of steroid hormones, in addition to its regulatory functions [29]. It has been shown that SIRT1 initiates the ROS scavenging pathway and maintains the normal function of granulosa cells, resists oxidative stress, maintains ovarian oxygen homeostasis, and plays a significant therapeutic role in PCOS and premature ovarian failure (POF) symptoms, mainly through the regulation of downstream proteins p53, FOXO1, FOXL2, CAT, and MnSOD.

Some drugs and other agonists can treat the dysregulation of oxygen homeostasis in ovarian follicles due to the FSH-induced upregulation of oxidative phosphorylation in granulosa cells, especially the abnormal metabolism of reactive oxygen species, by enhancing Sirt1 expression [30]. In addition, the authors found that SIRT1 also inhibited the phosphorylation of p66Shc, suppressed the activation of fibrogenic factors, improved the ovarian morphology, and reduced ovarian oxidative stress [31]. Li et al. also demonstrated that another agonist of SIRT1, oyster polypeptide, exerted a similar function to Ras in reducing oxidative stress [32]. Melatonin, one of the agonists of Sirt1, is also involved in processes related to follicular development and metabolism, and Shen et al. found that the inhibition of autophagy by the melatonin-PI3K-AKT-FOXO1 axis increased granulosa cell survival and also reduced oxidative damage by regulating SIRT1-FOXO1-ATG7-dependent autophagy [33]. Some non-coding RNAs also regulate Sirt1 expression. Carlomosti et al. found that miR-200c plays a significant role in oxidative stress by increasing the acetylation of p53 and FOXO1, decreasing catalase and manganese superoxide dismutase abundance, and increasing ROS production by targeting SIRT1 [34]. These studies demonstrate that SIRT1 in granulosa cells plays a regulatory role in ovarian oxidative stress through the deacetylation of downstream proteins to maintain the internal environment for normal ovarian function.

**Figure 3:**
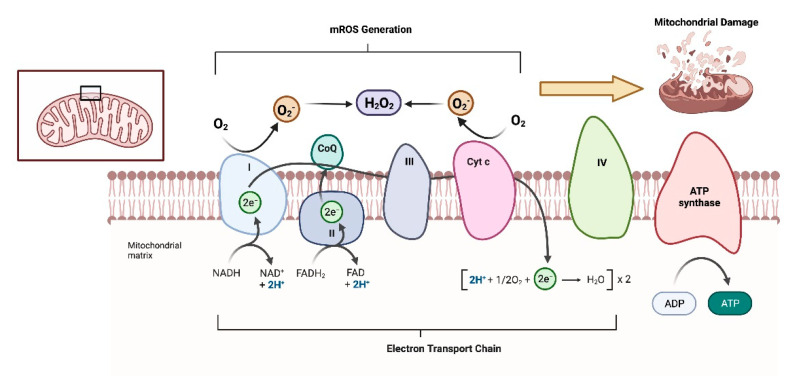
Energy metabolism and oxidative stress in granulosa cell mitochondria.

The transitional dependence on oxidative phosphorylation during follicular development inevitably causes elevated ROS, and excess ROS in the context of imbalanced ROS metabolism induces oxidative damage to mtDNA, the oxidation of specific amino acids, and lipid peroxidation. Consequently, these lead to abnormalities in granulosa oocyte communication in the preovulatory oocyte mass and, ultimately, apoptosis and death. In addition, studies have reported that excess ROS is one of the main causes of PCOS, significantly affecting the lifespan of ovarian tissues in women. Hyperglycemia leads to ROS formation, oxidative stress, and abdominal obesity. Persistent hormonal imbalance leads to multiple small sinus follicles and irregular menstrual cycles, which ultimately leads to female infertility [35]. In contrast, SIRT1 has a positive regulatory effect on the metabolism of ROS, which has significant implications for the maintenance of the ovarian lifespan and function and the regulation of follicular development processes (Figure 3).

### 3.2. Energy Metabolism and Mitochondrial Function

One of the main functions of granulosa cells is to transport metabolites and nutrients required for oocyte maturation, whereas the proliferation and differentiation of granulosa cells in the follicles occur simultaneously with oocyte maturation, and these two cells are metabolically coupled to each other [36]. As previously stated, pyruvate produced by the mitochondria of the granulosa cells of the ovarian follicle is the main source of energy during oocyte maturation due to the insufficient capacity of the oocyte to metabolize glucose. Granulosa cells metabolize glucose captured in the follicular microenvironment via glycolysis and supply their own pyruvate produced by glycolysis to the oocyte via gap junctions. Granulosa cells provide sufficient amounts of pyruvate, lactic acid, and nicotinamide adenine dinucleotide phosphate (NADPH) to oocytes through glycolysis and the tricarboxylic acid cycle. Oocytes produce the ATP required for oocyte maturation through the oxidative phosphorylation (OXPHOS) pathway [37]. While SIRT1, one of the proteins highly expressed in granulosa cells, has a relevant role in regulating energy metabolism and mitochondrial function, this regulation requires the involvement of an important energy sensor, AMPK.

Pan et al. found that the expression trends of AMPK/SIRT1 pathway-related genes in buffalo granulosa cells were consistent with those of glycolysis-related genes, and that SIRT1 activation increased the expression levels of both key glycolytic proteins and lactate production in granulosa cells, thus participating in the regulation of glycolysis and energy supply-related functions [38]. Yi et al. showed that melatonin activates PINK1/Parkin pathway-mediated mitochondrial autophagy, which is considered an important degradation pathway during energy stress and is essential for maintaining energy production, by enhancing the expression of SIRT1 in granulosa cells from patients with PCOS [39]. A subsequent study by Zheng et al. found that SIRT1, through the activation of Akt, is involved in the PI3K/Akt signaling pathway, thereby preventing mitochondrial membrane damage in ovarian granulosa cells, and thus may have the potential to treat mitochondrial damage in granulosa cells associated with PCOS [40]. Similarly, Zhang et al. improved ovarian energy metabolism by regulating the glycolytic pathway through the co-administration of Diane-35 and metformin in PCOS symptomatic rats and found that the treatment upregulated SIRT1 expression and improved the regulation of ovarian energy metabolism by regulating two key glycolysis-related rate-limiting enzymes, PKM2 and LDH-A [41]. Mihanfar et al. found that quercetin, also an agonist of SIRT1, was involved in regulating energy homeostasis via upregulating the expression of SIRT1 and AMPK, with beneficial effects on insulin resistance and hormonal indices [42]. Hisataka found that resveratrol increased SIRT1 expression levels and enhanced mitochondrial recovery from cryopreservation-induced damage in oocytes and embryos [43]. Itami et al. also found that resveratrol increased the level of ATP synthesis and improved the quality of porcine oocytes in early sinus follicles through the activation of SIRT1 during sow oocyte development [44]. Later studies found that short-term heat stress increased the expression level of heat shock protein 1, which induced the high expression of SIRT1 and phosphorylation of AMP-activated protein kinase, induced mitochondrial degradation and biogenesis, enhanced the mitochondrial membrane potential and ATP content of oocytes, and improved the ability of oocytes to develop into blastocysts [45]. Takeo et al. similarly found that resveratrol improved mitochondrial function and fertilization outcomes in bovine oocytes [46]. Daichi Sato et al. also found that the activation of SIRT1 by resveratrol in porcine oocytes increased the oocyte mitochondrial copy number and enhanced mitochondrial biosynthesis and degradation [47]. Emidio et al. found that SIRT1 combined with SIRT3 acted together to upregulate the levels of SOD2, PGC1α, mtTFA, and TOMM20, in addition to being subject to the activation of the SIRT1/AMPK axis involved in autophagy activation. SIRT1 and SIRT3 have been shown to act together to inhibit the increase in late glycosylation end-products induced by by-product methylglyoxal dependence, thereby attenuating the onset of glycolytic stress and oxidative stress [48].

Energy metabolism is one of the more unique and critical aspects of follicular development, because follicles rely heavily on the oxidative phosphorylation of granulosa cells, which inevitably produces metabolic by-products such as ROS and methylglyoxal, and the continuous accumulation of these substances can cause the functional impairment of mitochondria. In contrast, SIRT1 can regulate downstream proteins in mitochondria through its own deacetylation to reduce glycation stress and oxidative stress, and can also work together with AMPK to participate in autophagy and maintain basic mitochondrial functions. In addition, it has been found that AMPK/SIRT1 has significant therapeutic effects on insulin resistance in the ovaries, although further study is required to elucidate its specific mechanism of action.

## 4. Effect of SIRT1 on Premature Ovarian Failure and Ovarian Cancer

### 4.1. Premature Ovarian Failure

Although the average female life expectancy exceeds 70 years, ovarian gonadal tissue ages at a faster rate compared to other organs, with menopause (occurring at approximately 50 years of age) considered the end of the ovarian lifespan. The loss of ovarian folliculogenesis is accompanied by a significant decline in steroid hormone synthesis, and the hormonal imbalance is accompanied by an increased risk of cardiovascular disease, vasomotor changes, osteoporosis, and cognitive dysfunction; these symptoms can severely impact a woman’s quality of life in her later years [49]. Many studies in recent years have reported a prolonging effect of certain exogenous drugs, including melatonin, resveratrol, metformin, and rapamycin, as well as caloric restriction, on the ovarian lifespan, most of which involve the activation of SIRT1; these data therefore suggest an important role for SIRT1 in the regulation of the ovarian aging process.

Zhang et al. found that an abundance of melatonin in mouse follicular fluid decreased with age and that melatonin supplementation reversed the meiotic defect in senescent oocytes by activating SIRT1/SOD2 expression, improving aging oocyte quality, and returning the reproductive potential to senescent ovaries [50]. The rate of activation of resting primordial follicles into immature oocytes is a critical process that determines the ovarian reserves and reproductive lifespan. Zhang et al. found that SIRT1 promotes primordial follicle recruitment by directly regulating AKT and mTOR transcription through the modulation of the PTEN-PI3K-AKT and TSC1/2-mTOR signaling pathways, and, notably, this regulatory mechanism was independent of SIRT1 deacetylation [51]. In contrast, Nie et al. constructed a mouse model of premature ovarian failure by continuous ovulation overdrive, in which a significant reduction in the activity of the p16 and SIRT1/FOXO1 signaling pathways was found to be involved [52]. The adhesion proteins SA1 and SA2 are essential for normal chromosome segregation and DNA repair mediated by homologous recombination, and were found by Valerio et al. to be transcribed at high levels in follicles and to decrease significantly with age. SIRT1 is involved in the regulation of adhesion proteins by maintaining telomere integrity and thus regulating ovarian aging [53].

Modulation of Sirt1 expression by some drug treatments can play a role in regulating a delay in ovarian aging. Qin et al. treated 27-week-old female mice with metformin and assayed their ovarian functional activity after 6 months of rearing. The results showed that in the metformin-treated group, the expression of SIRT1 was significantly upregulated and these mice had higher E2 hormone levels and more stable estrous cycles. In addition, the levels of P16, 8-OHdG, 4-HNE, and p-rpS6 were reduced, suggesting that metformin may delay the ovarian aging process by inducing SIRT1 expression and reducing oxidative damage [54]. In their study, Zhang et al. noted that melatonin also acted as an agonist of AMPK/SIRT1 and significantly elevated the litter size in 24-week-old mice. These results suggest that melatonin delays ovarian aging and enhances fertility in mice via the MT1-AMPK-SIRT1 pathway [55]. Similarly, Hiroshi et al. found that ovarian aging was delayed, with increased mRNA expression and telomere length of LC3 in the treated group. These data show that melatonin delays ovarian aging through multiple mechanisms, including antioxidant effects, the maintenance of telomeres, the stimulation of SIRT1 expression, and enhancing ribosome function [56]. Ma et al. also found that melatonin protects against premature ovarian failure induced by tripterygium glycosides by activating SIRT1 [57]. Lee et al. found that resveratrol increased NAMPT expression in female Nothobranchius guentheri, thereby enhancing SIRT1 activity by increasing NAD+ abundance and exerting a protective effect against ovarian aging [58]. Similarly, Soliman et al. upregulated SIRT1 expression through resveratrol treatment, inhibiting NF-κB-induced inflammatory cytokines and restoring ovarian function in immature female Sprague-Dawley rats [59].

Previous studies have suggested that caloric restriction (CR) regulates many cellular functions and extends the lifespan of organisms. Liu et al. found that CR increased ovarian reserves by activating SIRT1 signaling in mice to inhibit follicle loss [60]. By generating SIRT1 knock-in mice, Long et al. enhanced fertility, preserved follicular reserves, and extended the ovarian lifespan by generating pure and heterozygous SIRT1 overexpressing mice with greater reproductive capacity compared to wild-type mice, in which SIRT1 was shown to activate FOXO3a and inhibit mTOR [61].

It is clear that SIRT1 plays an important role in the maintenance of ovarian tissue function and in the inhibition of ovarian aging, partly through the SIRT1/FOXO1 signaling pathway and through the deacetylation of other downstream proteins such as mTOR and AKT to reduce the accumulation of ROS. In the process of delaying ovarian aging, the role of AMPK/SIRT1 in regulating autophagy also plays an important role. In addition, the regulation of telomere integrity, adhesion proteins, and mitochondrial function by SIRT1 prolongs the ovarian tissue lifespan and also indirectly regulates ovarian reserves by participating in the life processes associated with primordial follicle activation, which are also highly correlated with the ovarian lifespan.

### 4.2. Ovarian Cancer

SIRT1 was initially identified as a longevity gene that plays a key role in the pathophysiology of metabolic and neurodegenerative diseases and is thought to contribute to the prevention of age-related diseases. SIRT1 has also been widely studied as a cancer gene in other tissues, especially in cancerous tumors of the liver, oral cavity, and bladder, and has been reported to have potential as a cancer therapeutic target. Due to the high abundance of SIRT1 in ovarian tissues, especially in granulosa cells, in addition to its involvement in the regulation of ovarian-related diseases such as PCOS, POI, POR, etc., some studies have implicated SIRT1 as a key transcription factor in the regulation of ovarian cancer.

In 2008, Zhang et al. found that SIRT1 was localized to the nucleus and cytoplasm of surface epithelial cells in OV2008 and C13 ovarian cancers and that it stimulated tumor cell migration through the deacetylation of cortical actin, supporting its potential as a new target for HDAC inhibitor-based therapies [62]. Shuang et al. similarly identified SIRT1 in plasmacytoid epithelial ovarian cancer (EOC) in higher abundance and found that its overexpression was significantly correlated with lower survival and higher chemoresistance, which may be a prognostic indicator of patient survival outcome [63]. Later, Duan et al. found that gerberoside effectively inhibited ovarian cancer cell growth in vitro and in vivo by increasing apoptosis. More importantly, it was found that gerberoside decreased SIRT1 expression, thereby attenuating the nuclear accumulation of β-catenin and subsequently inhibiting Wnt/β-catenin signaling, thus sensitizing cisplatin-resistant ovarian cancer cells to chemotherapy [64]. Miyamoto et al. found higher expression of SIRT1 in endometrioid, mucinous, and clear cell carcinomas compared to normal ovarian tissues and follicles, suggesting that high expression of SIRT1 is associated with cisplatin resistance in ovarian cancer cells and is one of the main reasons for poor prognosis in ovarian cancer patients [65]. These data reveal that the SIRT1 inhibitor, EX527, may have the potential to be a useful drug in the treatment of this malignancy [66]. An ovarian yolk cystic tumor is a rare but highly malignant and aggressive germ cell tumor. Kojima et al. found that the EZH2 and SIRT1 pathways are involved in the regulation of yolk cystic tumor differentiation and provide potential therapeutic options [67]. Zhang Yun et al. also demonstrated the knockdown of long-stranded non-coding RNA HOTAIR by repressing miR-138-5p-regulated EZH2 [68]. Xinjian Zhang et al. found that the deacetylation of SIRT1 promoted the nuclear accumulation of KLF4 in ovarian cancer cells and enhanced the binding of KLF4 to the CLDN5 promoter in the nucleus, contributing to the migration and invasion of malignant cancer cells [69].

The 5-year survival rate of ovarian cancer patients is only 47%, and cisplatin resistance remains one of the main reasons for poor prognosis in ovarian cancer patients. As such, there is an urgent need to develop new drugs for ovarian cancer. The above studies illustrate that SIRT1 is one of the major signaling molecules regulating ovarian cancer cells, and therefore drugs that inhibit SIRT1 can play a role in promoting autophagy in ovarian cancer cells. In particular, 15-deoxy-Δ12,14-prostaglandin J2 (15d-PGJ2) exhibits potent anticancer activity by inhibiting SIRT1, and it has been found that its derivatives (J11-C1 and J19) exhibit anticancer activity against ovarian cancer SKOV3 cells in ovarian tissues [70]. Subsequently, the authors identified a new SIRT1 inhibitor, MHY2245, which induces autophagy and inhibits energy metabolism in human ovarian cancer cells through the PKM2/mTOR pathway, providing new insights into the treatment of ovarian cancer. The authors used MHY2245 to treat SKOV3 ovarian cancer cells. MHY2245 significantly inhibited SIRT1 enzyme activity, decreased SIRT1 expression, promoted G2 cell cycle arrest in the M phase, and induced apoptotic cell death in SKOV3 cells by increasing the expression of apoptotic factors such as caspase-3 and Bax [71]. HDAC inhibitors can be used as a new class of anticancer agents to induce autophagy in cancer cells in different tissues. Jong et al. found that 15d-PGJ(2) induced apoptosis in different cancer cells through PPARγ non-dependent NF-κB and cystathione-dependent pathways, and exerted inhibitory effects on both SIRT1 and HDAC. Therefore, the development of deacetylase inhibitors may be key to the treatment of ovarian cancer [72].

Although the role of SIRT1 is essential in maintaining ovarian function, its mutation often causes cancer in ovarian tissues. High expression of SIRT1 in ovarian cancer cells promotes the migration, proliferation, and differentiation of cancer cells and it is highly associated with cisplatin resistance and poor prognosis in ovarian cancer patients. Therefore, it is of value to study the mechanism of action of SIRT1 in ovarian cancer and other related diseases, and to target SIRT1 in the development of appropriate drugs, such as specific inhibitors.

## 5. Potential for Regulating Intercellular Gap Junctional Communication

Intercellular communication plays a key role in the regulation of the cell cycle, cell survival, and proliferation. Gap junction proteins, which allow the passage of small molecules and nutrients through oocytes and granulosa cells, are essential for the growth and development of both. Granulosa cells participate in the regulation of oocyte development by providing critical metabolites and energy to the developing oocyte through gap junctional communication; oocytes can also regulate the proliferation and differentiation of granulosa cells by delivering growth factors, such as GDF9 and BMP15, through cell gap junctions [73]. In recent years, it has been found that the weakening of oocyte–granulosa cell gap junction communication is directly related to age and is accompanied by a significant downregulation of SIRT1 expression. This suggests that SIRT1 plays a crucial role in maintaining the normal function of oocyte–granulosa cell gap junction communication.

The transzonal projection (TZP) is a specialized filamentous pseudopod that mediates communication between the oocyte–granulosa cell gap junctions in the follicle and plays an important role in oocyte growth by maintaining bidirectional communication between the oocyte and the granulosa cell or oocyte. In 1998, Mary Jo Carabatsos et al. discovered that female mice with GDF9 deficiency underwent infertility caused by the failure of follicular development, and subsequently observed the degeneration of the connecting structures between granulosa cells and oocytes by ultrastructural (EM) and fluorescence microscopy analysis of follicles from GDF9-deficient mice. This was reinforced by a decrease in actin, the basic unit of the TZP that maintains bidirectional communication between oocytes and granulosa cells or oocytes. In a subsequent study [74], Chen et al. and Zhang et al. demonstrated that two important agonists of SIRT1, resveratrol and melatonin, respectively, maintain oocyte–granulosa cell gap junctional communication by increasing TZP activity, thus resisting ovarian dysfunction due to age and PCOS [75,76]. Although there is currently no evidence to support a direct role for SIRT1 in TZP production and the maintenance of function, it is plausible to assume that SIRT1 plays a role.

**Figure 4:**
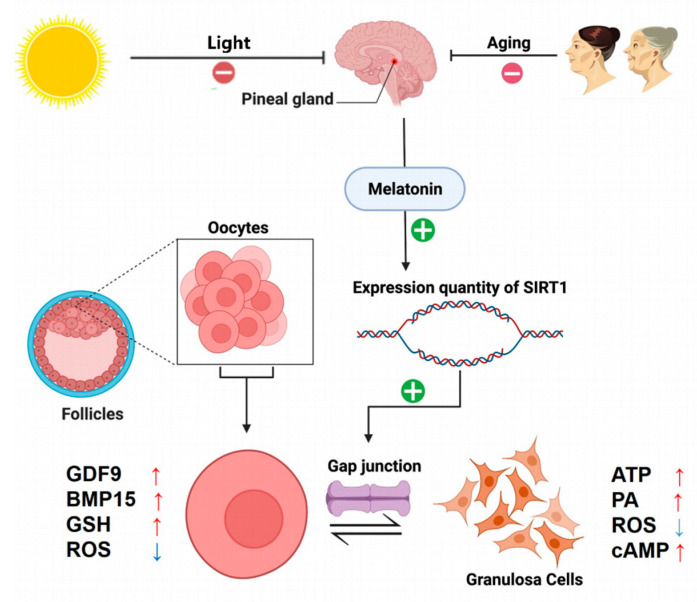
Regulatory effect of SIRT1 on cell gap junctional communication in ovarian granulosa cells.

SIRT1 can positively regulate the expression of Cx43 in the lung, heart, kidney, testis, intestine, and hypothalamus to restore the gap junctional communication between the oocyte and granulosa cells. SIRT1 can restore relevant cellular gap-linked communication in the lung, heart, kidney, testis, intestine, and hypothalamus by positively regulating Cx43 expression [77,78,79,80,81]. Since SIRT1 has the ability to migrate between the nucleus and cytoplasm, and is expressed in high abundance in ovarian tissues, the potential for SIRT1 to participate in oocyte–granulosa gap junction communication by regulating Cx43 expression in ovarian tissues needs to be investigated. In contrast, the association between SIRT1, Cx43, and photoperiod was first reported in early 2022 by Sriparna Pal et al. through the exploration of photoperiodic regulatory mechanisms. The pineal gland is innervated by the superior cervical ganglion, and the photoperiod regulates local Dio-2/Dio-3 conversion within the basal hypothalamic region of the brain in many vertebrate species to regulate seasonal reproduction. In this study, melatonin produced by the pineal gland served as an agonist for SIRT1. The authors found that the expression levels of SIRT1 and Cx43 were significantly increased in the ovaries of animals exposed to prolonged light exposure compared to the controls. The results of this study indicate that SIRT1 in the ovary may improve the fertility of women by upregulating the expression of Cx43 to enhance the gap junctions between ovarian granulosa cells and oocytes. This process, activated by melatonin, is also affected by the photoperiod [82] (as shown in Figure 4).

These data suggest that SIRT1 maintains the normal functioning of cell gap junctional communication pathways in different tissues by positively regulating the expression of Cx43. Since there are only a small number of studies that support the regulation of Cx43 by SIRT1 in the ovaries, the study of this mechanism is still in its infancy. Similarly, resveratrol and melatonin-related experiments have provided robust evidence that supports a role for SIRT1 in the maintenance of TZP activity through the regulation of actin. In conclusion, although the evidence for the direct regulation of oocyte–granule cell gap junction communication by SIRT1 is insufficient, the data available suggest that there is great potential and value in investigating this potential function further.

## 6. Conclusions and Future Prospects

The sirtuin family was discovered in yeast cells and has been studied by tens of thousands of scholars as a “longevity gene”, with the distant expectation of overcoming current limitations to the human lifespan [83]. In the past 50 years, significant progress has been made, and the mystery of the sirtuin family has been gradually unveiled. At a time when people were studying its function and mechanism of action in various tissues in order to prolong life, contrary to people’s expectations, the ovarian organ, one of the most “short-lived” organs in the human female body, could only maintain its function for less than half of the human lifespan and shows a precipitous decline in middle-aged women. It is worth celebrating that, with the unremitting efforts of many scholars and researchers, the sirtuin family from SIRT1 has been found to play a leading role in the regulation of ovarian-related functions, and the development of related drugs provides a clinical basis for the sirtuin family to maintain the regulation of ovarian function [84].

PCOS is a common endocrine and metabolic disorder in women of childbearing age, with an incidence rate as high as 10%. It is characterized by excessive androgen, rare ovulation, or anovulation. Insulin resistance and the related compensatory hyperinsulinemia are common features of PCOS [85]. Previous studies have reported that PCOS is closely related to the abnormal function of ovarian granulosa cells. Excessive oxidative stress and apoptosis of ovarian granulosa cells directly lead to abnormal follicular development and ovulation disorder in PCOS [86]; hyperinsulinemia and insulin resistance symptoms are caused by the mitochondrial dysfunction of ovarian granulosa cells in patients with PCOS [87]. The expression of Cx43 and Gap junction alpha 1 (GJA1) in the ovarian tissue of PCOS women is low, resulting in the obstruction of gap junction intercellular communication between granulosa cells and oocytes [88,89]. The granulosa cells of PCOS patients are also difficult to synthesize and secrete E2 smoothly [90]. Here, we found SIRT1, which is an effective cure for PCOS symptoms. In brief, SIRT1 promotes the proliferation of granulosa cells and inhibits apoptosis; metabolizes ROS as an antioxidant to maintain the internal environment; maintains mitochondrial function and the energy metabolism processes of granular cells; regulates the activation of primordial follicles to maintain ovarian function; maintains intrafollicular gap junction intercellular communication, and promotes the secretion of E2 by granulosa cells [91,92,93,94,95,96,97].

Because PCOS has relatively complex pathological symptoms and mechanisms, the previous treatment for PCOS patients can only temporarily relieve symptoms, accompanied by some side effects, and has no preventive effect [98]. In this review, we have revealed a potential treatment for PCOS infertility, rather than only one aspect. SIRT1 has a certain potential therapeutic effect on the comprehensive symptoms of PCOS patients. Of course, sufficient experiments are needed to verify this hypothesis. In short, this review aimed to provide the detailed mechanisms of these bioactive phytochemicals, and it is hoped to provide practical and reliable insights on the clinical application of PCOS.

## Data Availability

For the remaining data that may be relevant, the corresponding authors can be contacted.
